# ENDING AGEISM ISN'T ENOUGH: INFUSING AN INTERSECTIONAL LENS TO ADVANCE PRODUCTIVE AGING RESEARCH AND HEALTH

**DOI:** 10.1093/geroni/igac059.1107

**Published:** 2022-12-20

**Authors:** Ernest Gonzales

**Affiliations:** New York University, New York, New York, United States

## Abstract

This presentation integrates anti-racism, anti-ageism, and health equity lenses into the productive aging scholarship. Ageism and racism undermine population health and compromises choices to work and volunteer. These isms, among others, intersect and disproportionately impact populations of color and older adults; nonetheless, these oppressive systems create a culture of intergenerational conflict within the workplace and in general society. I will review key theoretical concepts and values in productive aging scholarship and how intersectionality as a framework has informed the development of new and important research questions for the field. This presentation will also analyze a variety of methodological approaches to examine productive aging and health inequities by race, ethnicity, gender, and age. A discussion on the implications for research, policy, and practice will conclude the presentation.

